# Cold-induced urticaria with a familial transmission: a case report and review of the literature

**DOI:** 10.1186/1752-1947-6-70

**Published:** 2012-02-20

**Authors:** Joe C Furr, Mukta Panda

**Affiliations:** 1 Department of Internal Medicine, University of Tennessee at Chattanooga, Chattanooga, TN, USA

## Abstract

**Introduction:**

Cryopyrin-associated periodic syndrome is a rare genetic disorder causing cold-induced urticaria, severe arthralgias, and (potentially) renal failure and hearing loss. Therapies that effectively control the symptoms and prevent the complications of this debilitating disorder are now available, making recognition of this disease important.

**Case presentation:**

A 60-year-old Caucasian woman presented with complaints of rash and joint pains to a general medicine clinic. Her history showed that her symptoms were linked to cold exposure, but the results of a cold stimulation time test were negative. Several generations of her family had similar symptoms.

**Conclusions:**

This case highlights the importance of considering cryopyrin-associated periodic syndrome in the differential diagnosis of cold-induced urticaria. Several medications targeting interleukin-1-beta are available, providing significant relief from symptoms and improvement in quality of life in affected patients.

## Introduction

Cryopyrin-associated periodic syndrome (CAPS) is a rare inherited inflammatory disorder with a unique pathophysiology related to overproduction of interleukin-1-beta (IL-1β). CAPS consists of three syndromes: the familial cold autoinflammatory syndrome (FCAS), Muckle-Wells syndrome (MWS), and neonatal onset multisystem inflammatory disease (NOMID) (also known as the chronic infantile neurologic, cutaneous, articular [CINCA] syndrome). These disorders share a number of phenotypic features and represent a continuum of disease severity; FCAS is at the milder end, NOMID/CINCA syndrome is at the more severe end, and MWS is an intermediate form. Although they are described as distinct disorders, there is some overlap of symptoms among them. Inflammasome overactivation leading to overproduction of IL-1β underlies all of these disorders, most often due to autosomal dominant inheritance of missense mutations in the gene coding for cryopyrin [1].

## Case presentation

**Figure 1:**
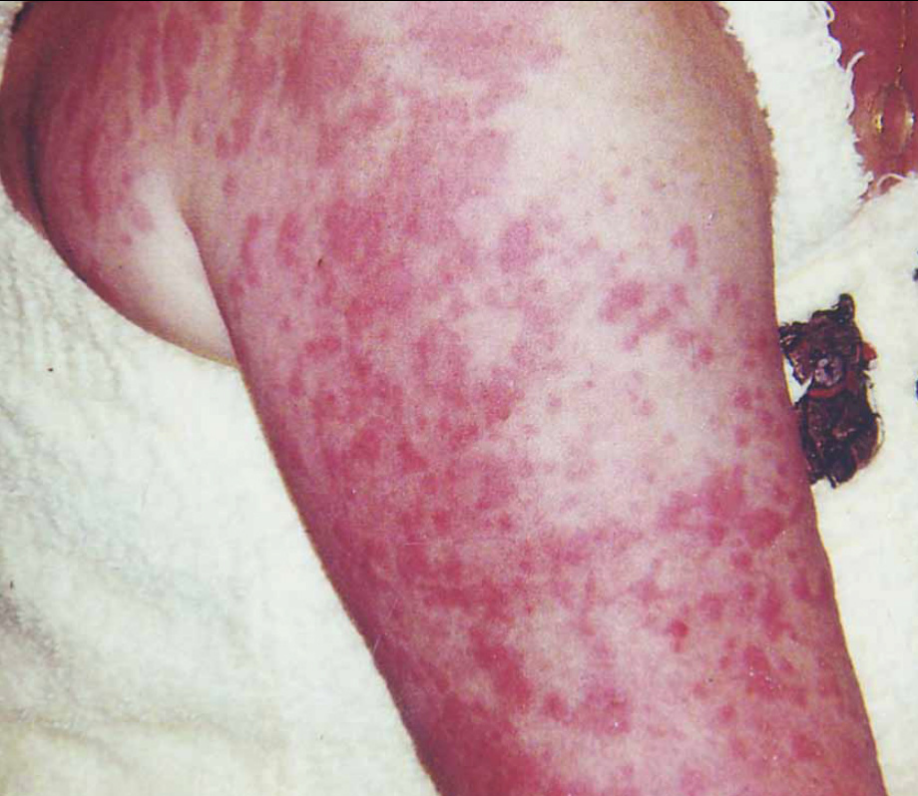
**Rash in cryopyrin-associated periodic syndrome (CAPS)**. The rash seen in CAPS has an urticaria-like appearance. Our patient brought this photograph with her to her first appointment with us.

A 60-year-old Caucasian woman presented to her primary care physician with complaints of chronic rash and joint pains associated with exposure to cold. She described episodes of fever and chills associated with an erythematous maculopapular rash (Figure 1), headache, and debilitating joint pains precipitated by cold. These symptoms had been present since childhood and had worsened over time. She reported that other family members had experienced similar symptoms, reportedly as far back as five generations. She had seen several physicians for the evaluation of her symptoms, which had been ascribed to a variety of disorders, including systemic lupus erythematosus and acquired cold urticaria. Her symptoms had been treated with steroids and antihistamines, which provided little or no relief. A cold stimulation time test was performed and did not produce an urticarial wheal. On the basis of her symptoms, family history, and negative response to a cold time stimulation test, a working diagnosis of CAPS was made. This led to her enrollment in a clinical trial of an IL-1β monoclonal antibody, providing near-complete relief from her debilitating symptoms. Genetic testing later confirmed a diagnosis of FCAS.

## Discussion

The cold urticaria syndromes are a heterogeneous group of disorders characterized by the development of inflammation following cold exposure. Cold urticaria has a variety of causes and can be classified broadly into acquired and familial cold forms. Acquired cold urticaria may occur as a primary disorder or secondary to another process. Common secondary causes include cryoglobulinemia, infectious diseases (syphilis, rubeola, varicella, hepatitis, and infectious mononucleosis), or certain drugs (penicillin, oral contraceptives, and angiotensin-converting enzyme inhibitors) [2]. The history of response to cold exposure may give clues to whether a cold-induced urticaria is an acquired cold urticaria or is due to a familial cause. In contrast to the majority of acquired cold urticarias, CAPS is characterized by a delayed response to cold exposure. An early age of onset as well as the presence of fever and symptoms due to complications of CAPS may also help differentiate it from acquired cold urticarias [2].

A key diagnostic test in the evaluation of cold-induced urticaria is the cold stimulation time test. In this study, a cold stimulus, such as an ice cube in a plastic bag, is applied for five minutes

to the patient's skin, which then is allowed to rewarm. The test is considered positive if a coalescent wheal forms. It then can be repeated with shorter times of cold stimulation to better define the degree of sensitivity. If the test is negative with a five minute application, it may be repeated with a 10-minute application [2,3]. The cold stimulation time test does not typically form an urticaria wheal in patients with CAPS as its symptoms, unlike those of acquired cold urticaria, are not mediated by histamine release.

CAPS is a systemic inflammatory disorder occurring as a result of autosomal dominant or *de novo *mutations in the gene *NLRP3 *(*CIAS1*). Mutations in this gene lead to production of an altered form of the protein, cryopyrin. Cryopyrin, as a member of the NALP3 inflammasome, activates caspase-1, which in turn activates IL-1β. These changes are thought to cause a gain-of-function effect of the NALP3 inflammasome, leading to overproduction of IL-1β [4].

Inflammasome activation and IL-1β overproduction have been implicated in the pathogenesis of a number of diseases, including type 2 diabetes, gout, and rheumatoid arthritis [5]. A recent trial of IL-1β blockade in people with type 2 diabetes showed improvements in glycemic control and pancreatic β-cell function [6], and additional studies to better define the potential role of IL-1β blockade in the treatment of diabetes are ongoing. CAPS is a systemic inflammatory disorder affecting a number of organ systems. Most commonly involved is the skin, and a maculopapular, urticaria-like, and usually nonpruritic rash is typical. Skin lesions are seen in the first six months of life in nearly all patients [7] and at birth in nearly two thirds of patients with CINCA/NOMID [8]. The onset of symptoms following cold exposure is delayed an average of two and a half hours and duration is up to 12 hours in patients with FCAS, and attacks in MWS last about one to two days [7,9]. The correlation of symptoms with cold exposure is less consistent in MWS than in FCAS. CAPS may also affect the musculoskeletal, renal, neurologic, and ocular systems. Joint symptoms range from arthralgias in FCAS to severe joint deformation in CINCA/NOMID. Renal involvement is often seen in patients with MWS and CINCA/NOMID, and elevations in serum amyloid A levels potentially lead to renal amyloidosis in about 25% of patients with MWS [9]. Ocular involvement consists mainly of conjunctivitis and episcleritis, although blindness may occur in patients with CINCA/NOMID. Progressive sensorineural hearing loss is seen in about 60% of patients with MWS [9].

Advances in the understanding of the pathogenesis of CAPS resulted in the use of therapies targeting IL-1β in the treatment of this disorder. First developed among these agents was anakinra, a recombinant human IL-1 receptor antagonist. Originally approved for use in rheumatoid arthritis, it reduces the inflammation associated with CAPS [10,11]. Anakinra has been shown to improve symptoms of CAPS and also may reverse or stabilize hearing loss and renal amyloidosis due to CAPS [12]. The symptomatic improvements seen with anakinra, however, are contingent upon continued daily administration, and withdrawal leads to a re-emergence of symptoms [10,11].

Rilonacept, a fusion protein of the extracellular IL-1 receptor and the Fc portion of human IgG1, is a newer alternative therapy. This agent traps IL-1, binding it and preventing it from interacting with the IL-1 receptor. Its high affinity for IL-1β allows once-weekly dosing [13]. The safety and efficacy of rilonacept were evaluated in two randomized, placebo-controlled trials, in which rilanocept therapy resulted in rapid and sustained improvement of symptoms. Serum amyloid A and C-reactive protein levels also were reduced with rilonacept. The most common adverse events seen with rilonacept therapy are injection site reactions and upper respiratory infections [14].

Canakinumab is the newest medication released for the treatment of CAPS. This agent is a fully human anti-IL-1β monoclonal antibody with a plasma half-life of 28 to 30 days, allowing dosing once every eight weeks. In a randomized, double-blind, placebo-controlled trial, canakinumab administration led to rapid and sustained reduction of inflammatory symptoms and normalization of serum amyloid A levels in patients with CAPS. Canakinumab was well tolerated and caused few injection site reactions, although the incidence of suspected infection was higher versus placebo [15].

## Conclusions

CAPS should be considered in the differential diagnosis of any patient presenting with cold-induced urticaria-like symptoms. As in our case, symptoms due to CAPS may be attributed erroneously to a variety of disorders, such as acquired cold urticaria or other rheumatologic disorders like systemic lupus erythematosus. A thorough history, focusing on the timing of symptom onset in relation to cold exposure and the presence of the other family members with similar symptoms, helps identify affected individuals. Several medications targeting IL-1β are available, providing significant relief from symptoms and improvement in quality of life in patients with CAPS.

## Abbreviations

CAPS: cryopyrin-associated periodic syndrome; CINCA: chronic infantile neurologic, cutaneous, articular; FCAS: familial cold autoinflammatory syndrome; IL-1β: interleukin-1-beta; MWS: Muckle-Wells syndrome; NOMID: neonatal onset multisystem inflammatory disease.

## Consent

Written informed consent was obtained from the patient for publication of this case report and any accompanying images. A copy of the written consent is available for review by the Editor-in-Chief of this journal.

## Competing interests

The authors declare that they have no competing interests.

## Authors' contributions

JCF performed the literature review and co-authored the manuscript. MP co-authored the manuscript. Both authors read and approved the final manuscript.
